# Changes in various metabolic parameters in blood and milk during experimental *Escherichia coli* mastitis for primiparous Holstein dairy cows during early lactation

**DOI:** 10.1186/2049-1891-5-47

**Published:** 2014-10-17

**Authors:** Kasey M Moyes, Torben Larsen, Peter Sørensen, Klaus L Ingvartsen

**Affiliations:** Department of Animal and Avian Sciences, University of Maryland, 142 Animal Sciences Building, MD 20742-2311, 20910 College Park, MD USA; Department of Animal Science, Faculty of Science and Technology, Aarhus University, Tjele, 8830 Denmark

**Keywords:** Cow, Early lactation, *Escherichia coli*, Metabolism

## Abstract

**Background:**

The objective of this study was to characterize the changes in various metabolic parameters in blood and milk during IMI challenge with *Escherichia coli* (***E. coli***) for dairy cows during early lactation. Thirty, healthy primiparous Holstein cows were infused (h = 0) with ~20-40 cfu of live *E. coli* into one front mammary quarter at ~4-6 wk in lactation. Daily feed intake and milk yield were recorded. At –12, 0, 3, 6, 12, 18, 24, 36, 48, 60, 72, 96, 108, 120, 132, 144, 156, 168, 180 and 192 h relative to challenge rectal temperatures were recorded and quarter foremilk was collected for analysis of shedding of *E. coli*. Composite milk samples were collected at -180, -132, -84, -36, -12, 12, 24, 36, 48, 60, 72, 84, 96, 132 and 180 h relative to challenge (h = 0) and analyzed for lactate dehydrogenase (**LDH**), somatic cell count, fat, protein, lactose, citrate, beta-hydroxybutyrate (**BHBA**), free glucose (**fglu**), and glucose-6-phosphate (**G6P**). Blood was collected at -12, 0, 3, 6, 12, 18, 24, 36, 60, 72, 84, 132 and 180 h relative to challenge and analyzed for plasma non-esterified fatty acids (**NEFA**), BHBA and glucose concentration. A generalized linear mixed model was used to determine the effect of IMI challenge on metabolic responses of cows during early lactation.

**Results:**

By 12 h, *E. coli* was recovered from challenged quarters and shedding continued through 72 h. Rectal temperature peaked by 12 h post-challenge and returned to pre-challenge values by 36 h post-IMI challenge. Daily feed intake and milk yield decreased (*P* <0.05) by 1 and 2 d, respectively, after mastitis challenge. Plasma BHBA decreased (12 h; *P* <0.05) from 0.96 ± 1.1 at 0 h to 0.57 ± 0.64 mmol/L by 18 h whereas concentration of plasma NEFA (18 h) and glucose (24 h) were significantly greater, 11 and 27%, respectively, after challenge. In milk, fglu, lactose, citrate, fat and protein yield were lower whereas yield of BHBA and G6P were higher after challenge when compared to pre-challenge values.

**Conclusions:**

Changes in metabolites in blood and milk were most likely associated with drops in feed intake and milk yield. However, the early rise in plasma NEFA may also signify enhanced adipose tissue lipolysis. Lower concentrations of plasma BHBA may be attributed to an increase transfer into milk after IMI. Decreases in both milk lactose yield and % after challenge may be partly attributed to reduced conversion of fglu to lactose. Rises in G6P yield and concentration in milk after challenge (24 h) may signify increased conversion of fglu to G6P. Results identify changes in various metabolic parameters in blood and milk after IMI challenge with *E. coli* in dairy cows that may partly explain the partitioning of nutrients and changes in milk components after IMI for cows during early lactation.

## Background

During early lactation (i.e. 0-8 wk in milk), the homeorhetic mechanisms associated with hormonal changes, as well as changes in the nervous system and immune system, shift the partitioning of nutrients from peripheral tissues towards the synthesis of milk. This massive re-partition has been identified as a major contributor to the high risk of disease at this time [1]. Mastitis, an inflammation of the mammary gland, is the most costly of all diseases and occurs more frequently after parturition [2, 3]. The innate immune response patterns to major mastitis-causing pathogens (e.g. *E. coli*, *Streptococcus uberis* and *Staphylococcus aureus*) have been well-documented [4–6] but the characterization of the metabolic responses in dairy cows during an IMI are not fully understood.

Most studies have focused on the effect of metabolic status on immune response for dairy cows [1, 7, 8]. During mastitis, the immunometabolic responses primarily focus on the transcription-level responses in liver and mammary tissue [9–11]. Previous work indicates that the ability of the liver to metabolize fatty acids is reduced and key genes associated with metabolic processes are down-regulated after intramammary *E. coli* challenge [12] as well as after intramammary endotoxin challenge [13]. Furthermore, changes in circulating non-esterified fatty acids (**NEFA**), beta-hydroxybutyrate (**BHBA**) and glucose, prior to decreases in feed intake and milk production, during an IMI in dairy cows have been reported [14–16]. To our knowledge, changes in free glucose (**fglu**) and glucose-6-phosphate (**G6P**) in milk during mastitis in relation to changes in circulating metabolites and other milk components has not been elucidated. The mammary gland primarily relies on circulating glucose for the synthesis of lactose, a disaccharide composed of the monosaccharides D-glucose and D-galactose [17]. Other fates of glucose in the mammary gland include the conversion to G6P for the synthesis of galactose [18]. Characterizing the metabolic responses of fglu and G6P in relation to other metabolic components during inflammation may further elucidate the partitioning of nutrients and changes in milk composition that occur during mastitis. The objective of this study was to characterize the changes in various metabolic parameters in blood and milk during IMI with *E. coli* for dairy cows during early lactation.

## Methods

The experiment was carried out at the cattle research facilities at Department of Animal Science, Aarhus University. Experimental procedures involving animals were approved by the Danish Animal Experiments Inspectorate and complied with the Danish Ministry of Justice Laws concerning animal experimentation and care of experimental animals.

### Animals, experimental design and sample collection

Thirty primiparous Holstein cows at ~4-6 wk in lactation were used for this study. Only healthy cows not treated for any clinical signs of disease before the study period were included. Details on animal housing, total mixed ration fed and refused, treatment, preparation and infusion of *E. coli* and clinical examinations have been previously described [19, 20]. Briefly, eligible cows were considered healthy and free of mastitis-causing pathogens based on body temperature, white blood cell count, glutaraldehyde test, California Mastitis Test (Kruuse, Marslev, Denmark) and bacteriological examinations of aseptic quarter foremilk samples prior to the start of the study period. Using the portable DeLaval Cell Counter (DeLaval, Tumba, Sweden), the front quarter with the lowest somatic cell count (**SCC**; <27,000 cells/mL) was used for *E. coli* infusion.

Cows were housed and fed in individual straw-bedded tie-stalls, had free access to water, and were milked twice at 0600 and 1700 h. Cows averaged 27.5 ± 5.5 kg milk/d at the start of the trial. Cows were fed a standard total mixed ration for lactating cows ad libitum twice at 0800 and 1530 h. Daily feed intake and milk yield were recorded throughout the study period. Orts were collected in the mornings (~0800 h). To clarify, IMI challenge occurred after the afternoon milking, and therefore, d = 0 was calculated from milk yield and feed intakes from -36 to -12 h prior to IMI challenge where -12 h represents the morning relative to challenge.

All cows were infused with ~20-40 cfu of live *E. coli* (Danish field isolate k2bh2) into one front mammary quarter immediately following the afternoon milking (h = 0). The IMI challenge was imposed in the same year (i.e. 2007) and stage of lactation but in 4 different blocks: May (n = 8), June (n = 7), August (n = 8) and September (n = 8). Rectal temperature was recorded at -12, 0, 3, 6, 12, 18, 24, 48, 60, 72, 84, 96, 108, 120, 132, 144, 156, 168, 180 and 192 h relative to IMI challenge. Composite milk samples were collected relative to IMI challenge (h = 0) during the morning milking period at -180, -132, -84, -36, and -12 h, at each milking after challenge at 12, 24, 36, 48, 60, 72, 84, 96 h, and during the morning milking period at 132 and 180 h. Aseptic quarter foremilk samples were collected from challenged quarters at -12, 0, 3, 6, 12, 18, 24, 48, 60, 84, 96, 108, 120, 132, 144, 156, 168, 180 and 192 h relative to IMI challenge. One day prior to IMI challenge, sterile Micro-Renathane polyvinyl catheters were inserted into the jugular vein and flushed with a sterile 0.9% NaCl solution containing 50 IU Na-heparin (Loevens Kemiske Fabrik, Ballerup, Denmark). Blood was collected at -12, 0, 3, 6, 12, 18, 24, 36, 60, 84, 132 and 180 h relative to IMI challenge. For a subset of cows (n = 16), liver biopsies were collected at -144, 12, 24 and 192 h relative to IMI challenge for gene expression profiling and results are reported elsewhere [12]; and a mammary biopsy was collected at 24 and 192 h relative to IMI challenge for gene expression analysis using a minimally invasive biopsy technique [19]. After the mammary biopsies had been collected (i.e. 24 and 192 h post-biopsy), cows were administered a prophylactic antibiotic treatment against infection with Gram-positive bacteria by intramuscular injection of 30 mL of Penovet® vet (300,000 IE benzylpenicillinprocain/ml; Boehringer Ingelheim Danmark A/S,Copenhagen, Denmark). No other antibiotic therapy was administered after IMI challenge for all cows, regardless of biopsy.

### Sample analysis

Composite milk samples were analyzed for fat, protein, lactose, citrate and SCC (cells/mL) using a CombiFoss 4000 (Foss Electric A/S, Hillerød, Denmark) and BHBA, lactate dehydrogenase (**LDH**), N-acetyl-β-D-glucosaminidase (**NAGase**) and alkaline phosphatase activity (**ALP**) were analyzed according to methods previously described [21–23]. Free glucose and G6P were analyzed by an enzymatic-fluorometric method as described by Larsen [24]. Quarter foremilk was analyzed for SCC and quantification of *E. coli* (cfu/mL) as previously described [19].

Plasma was harvested following centrifugation at 2,000 × *g* for 20 min at 4°C and stored at -18°C until further analysis. All plasma components were analyzed for NEFA, BHBA and glucose using an autoanalyzer, ADVIA 1650® Chemistry System (Siemens Medical Solution, Tarrytown, NY, USA) according to methods described by Bjerre-Harpøth et al. [21].

### Statistical analysis

Plasma NEFA and BHBA were normalized by natural log (**ln**) transformation and SCC and shedding of *E. coli* were log_10_ transformed for statistical analyses. Yields for all milk components were calculated at each milking. Data were analyzed via a generalized linear mixed model using the MIXED procedure of SAS, version 9.3 [25] with the repeated measure of time (i.e. hour) relative to IMI challenge (h = 0). Using the MIXED procedure of SAS, combined biopsy had no effect (*P* <0.05) on any of the metabolic parameters in blood and milk for this study and was, therefore, left out of the final model. The random effect of cow within block was used as the error term in the REPEATED statement with compound symmetry (**CS**) as the covariance structure. The model was used to determine the effect of IMI challenge on metabolic and immune responses in blood and milk for cows in early lactation. The class variables included cow, block and time relative to IMI challenge. Degrees of freedom were estimated with the Kenward-Roger specification in the model statements. Separation of least square means (**LSM**) for significant effects was accomplished using the Tukey’s option within the MIXED procedure of SAS. Statistical differences were declared as significant and highly significant at *P* <0.05 and *P* <0.01, respectively. Trends towards significance are discussed at *P* <0.10. Plasma NEFA and BHBA were back-transformed for presentation in figures.

## Results and discussion

### Indicators of infection and immune response

**Figure 1:**
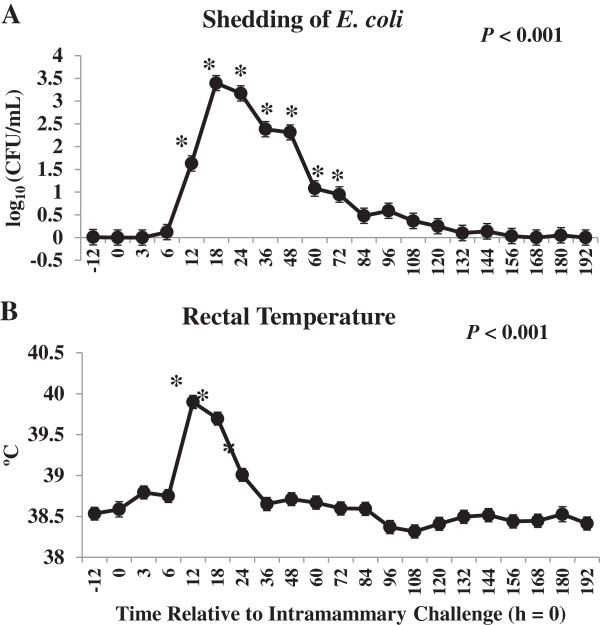
**Colony forming units (cfu) of** ***Escherichia coli*** ***(E. coli)/*** **mL of mammary secretion (A) and rectal temperature (B) after intramammary challenge with** ***Escherichia coli*** **(h** **=** **0)** **for 30 primiparous Holstein cows during early lactation.** *Differences (*P* <0.05) when compared to h = 0.

The bacterial counts of *E. coli* (A) and rectal temperature (B) relative to IMI challenge are shown in Figure 1. By 12 h, *E. coli* was recovered from challenged quarters and shedding continued through 72 h similar to those of others [26, 27]. Rectal temperature returned to pre-challenge values by 36 h post-IMI challenge as observed by Scaletti and Harmon [27]. These findings are consistent with signs of experimental *E. coli* mastitis and confirmed the model system.

**Figure 2:**
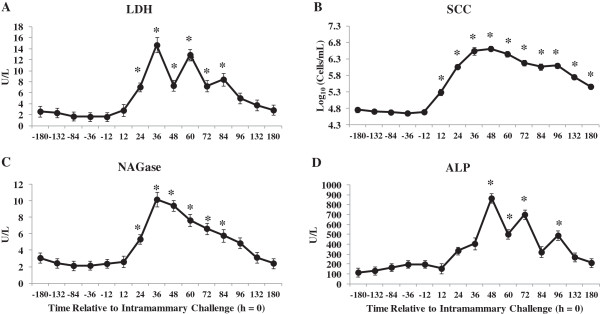
**Composite milk concentrations of lactate dehydrogenase (LDH;A), somatic cell count (SCC; B),** **N-** **acetyl** **-** **β** **-** **D** **-** **glucosaminidase (NAGase; C) and alkaline phosphatase (ALP; D) at time points relative to intramammary challenge with** ***Escherichia coli*** **(h** **=** **0)** **in 30 primiparous Holstein cows during early lactation.** *Differences (*P* <0.05) when compared to h = 0.

Composite milk LDH (A), SCC (B), NAGase (C) and ALP (D) concentrations were greater after IMI challenge with *E. coli* (Figure 2). During an IMI challenge, a cascade of changes occur including increased LDH, ALP and NAGase activity in milk associated with infiltrating neutrophils and resident macrophages [28, 29]. These indigenous enzymes are accurate real-time indicators for detecting mastitis on-farm when compared to composite SCC or bacterial culture [28–30].

### Cow-level and metabolic responses

**Figure 3:**
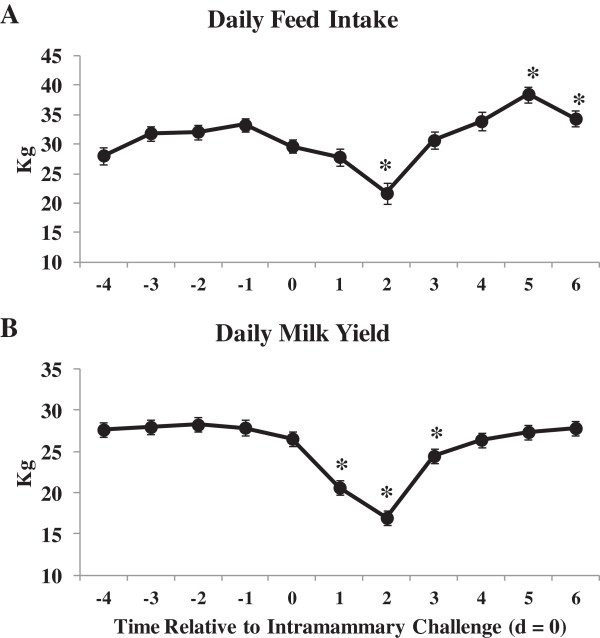
**Daily feed intake (as fed; A) and daily milk yield (B) for 30 primiparous Holstein cows during early lactation relative to intramammary challenge with** ***Escherichia coli*** **(h** **=** **0)** **.** *Differences (*P* <0.05) when compared to d = 0 (d = 0 includes -36 to -12 h relative to challenge).

Changes in daily feed intake (as fed; A) and milk yield (B) relative to IMI challenge are shown in Figure 3. To clarify, d = 0 reflects the daily feed intake and milk yield from the 24 h period prior to IMI challenge (i.e. -36 to -12 h relative to IMI challenge). Day = 1 reflects -12 to 12 h post-IMI challenge. Daily milk yield decreased 23% from d = 0 to d = 1 and 36% by d = 2 whereas feed intake was not significantly reduced until 2 d post-IMI challenge from 29.7 kg at d = 0 to 21.7 kg by d = 2. Feed intake and milk yield returned to pre-challenge values by 3 and 4 d post-IMI challenge, respectively. As lactation progressed, feed intake continued to increase as normally observed during early lactation [31]. Decreases in feed intake and milk yield have been previously shown for cows experimentally challenged with *E. coli* during early lactation [27]. Multiple local and systemic factors, i.e. production of cytokines and the changing hormonal environment, contribute to reduced feed intake and milk production observed during an IMI [32] and most likely explain the majority of variation in blood and milk metabolites for this study.

**Figure 4:**
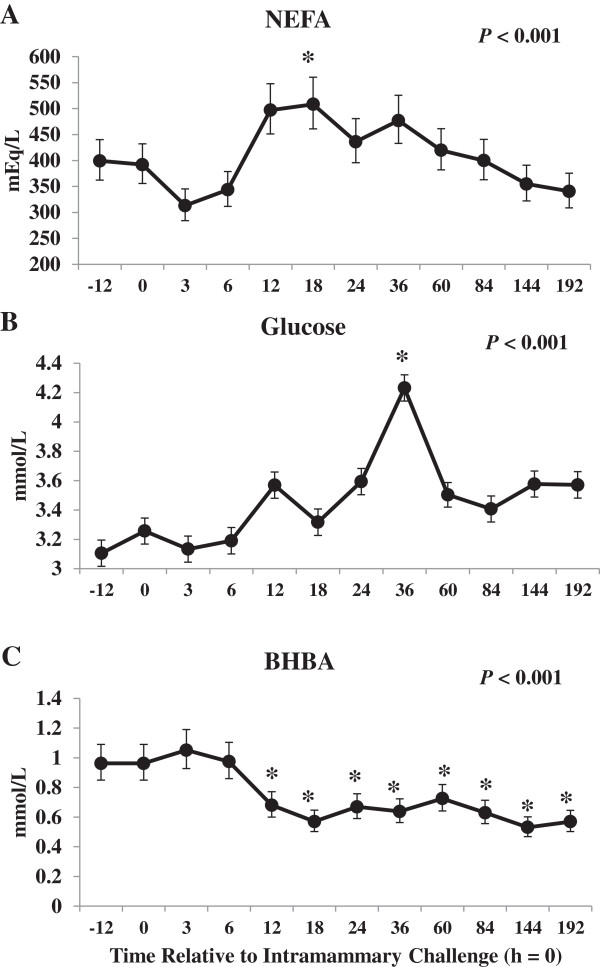
**The effect of intramammary challenge with** ***Escherichia coli*** **(h** **=** **0)** **on concentration of plasma non** **-** **esterified fatty acids (NEFA; A)**, **glucose (B) and beta**-**hydroxybutyrate (BHBA; C) in 30 primiparous Holstein cows during early lactation.** Samples collected at -12, 12, 36, 60, 84, 132 and 180 h were collected prior to morning feeding. *Differences (*P* ≤0.05) when compared to h = -12.

Changes in concentration of plasma NEFA (A), glucose (AB) and BHBA (C) relative to IMI challenge are shown in Figure 4. Blood samples collected prior to the morning feeding included -12, 12, 36, 60, 84, 132 and 180 h relative to IMI challenge. Plasma NEFA increased 11% by 18 h, after the morning feeding, but no other time points differed from pre-challenge values throughout the study period. Although changes in plasma NEFA may be primarily attributed to changes in feed intake, increases in plasma NEFA may also be associated with enhanced lipolysis in adipose tissue, regardless of changes in feed intake, which has been proposed to be the primary source of elevated NEFA in blood during inflammation [33]. Steiger et al. [34] showed increased NEFA in blood following a prolonged low-dose intravenous (**IV**) lipopolysaccharide (**LPS**) infusion in non-lactating heifers. Similar results were reported after intramammary challenge with LPS for both primiparous and multiparous cows at 7 days in milk [14] and after IV infusion of LPS for multiparous cows in mid-lactation [16]. These results contradict those of Waldron et al. [15] where plasma NEFA decreased after intramammary LPS infusion for multiparous cows in early lactation as well as Moyes et al. [7] where no change in plasma NEFA was observed after IMI challenge with *Streptococcus uberis* for multiparous cows in mid-lactation. Our results, and those of others, indicate that plasma NEFA increase, regardless of stage of lactation or parity, in response to both live bacteria reported here (i.e. *E. coli*) and LPS administered either intramammary [16] or IV [34].

Plasma glucose was greater at 24 and 36 h when compared to -12 h (Figure 4B) where plasma glucose was 26.6% greater at 36 when compared to -12 h relative to IMI challenge. Increases in plasma glucose are primarily attributed to changes in feed intake and reduced demand for lactose synthesis in the mammary gland in response to IMI challenge. However, Steiger et al. [34] observed increases in plasma glucose after IV LPS infusion in non-lactating heifers indicating that hyperglycemia is independent of changes in milk production.

The mechanisms regulating glucose homeostasis during IMI are unclear and the primary theories are 1) changes in feed intake, 2) increased circulating glucocorticoids observed during infection [16, 35], 3) decreased lactose synthesis in the mammary gland, 4) increased hepatic lactate recycling via the Cori cycle [16, 36], 5) increased glycogenolysis in peripheral tissues [34, 37] and/or 6) increased hepatic gluconeogenesis [36]. Increases in glucocorticoids observed after infection are associated with increased adipose tissue lipolysis, increased hepatic gluconeogenesis and inhibition of insulin sensitivity in skeletal muscle [38]. Changes in plasma glucocorticoid concentrations were not assessed for this study and the contribution to changes in plasma glucose are unknown. Hyperglycemia has been reported in sheep [36] and in non-lactating dairy heifers [34] after inflammation and therefore decreases in milk lactose synthesis may not be the only factor explaining increases in plasma glucose at this time. Furthermore, glycogen stores are largely depleted during early lactation [39] and increased glycogenolysis unlikely explains changes in glucose supply based on transcriptional responses in liver for this study [12]. A down-regulation of key genes associated with hepatic gluconeogenesis was observed in liver tissue for this study by 24 h post-IMI challenge [12] including phosphoenolpyruvate carboxykinase 1 (*PCK1*; -8.2-fold change versus pre-IMI challenge) and glucose-6-phosphatase (*G6PC*; -1.7-fold change). However, an up-regulation of both lactate dehydrogenase A (*LDHA*; 1.2-fold change) and B (*LDHB*; 1.1-fold-change) were observed in liver at 24 h post-IMI challenge. Both *LDHA* and *LDHB* code for functional LDH, the enzyme responsible for the reversible conversion of lactate to pyruvate, and supports the theory of a potential increase in lactate recycling via the Cori cycle during IMI challenge [16, 36].

**Figure 5:**
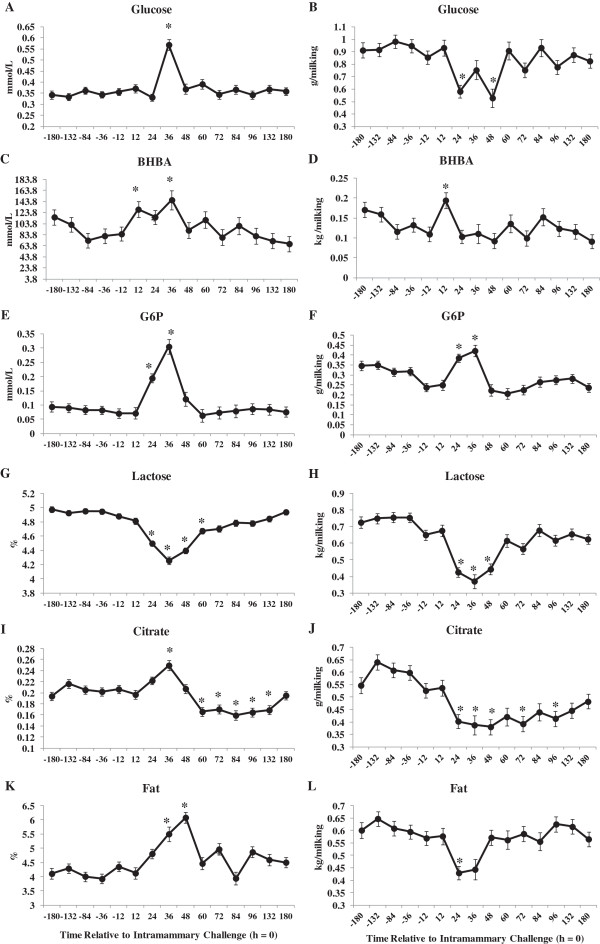
**The effect of intramammary challenge with** ***Escherichia coli*** **(h** **=** **0)** **on concentration and yield of milk glucose (A and B, respectively),** **BHBA (C and D, respectively),** **glucose** **-** **6** **-** **phosphate (G6P; E and F, respectively),** **lactose (G and H, respectively),** **citrate (I and J, respectively) and fat (K and L, respectively) in 30 primiparous Holstein cows during early lactation.** Samples collected at -12, 12, 36, 60, 84, 132 and 180 h were collected prior to morning feeding. *Differences (*P* ≤0.05) when compared to h = -12.

Plasma BHBA decreased by 12 h post-IMI challenge when compared to pre-challenge levels (Figure 4C). Other studies have reported decreased circulating concentrations of BHBA during an intramammary challenge with LPS for lactating dairy cows [14, 15, 34]. During inflammation, decreases in BHBA in blood are a consequence of either 1) increased blood glucose; 2) changes in BHBA supply via reduced rumen motility [37, 40]; 3) impairment of hepatic ketogenesis [34, 41]; or 4) a combination of the above. Transcriptional profiling of liver tissue indicated a down-regulation of genes associated with hepatic ketogenesis [12] including 3-hydroxy-3-methylglutaryl-CoA synthase 1 (*HMGCS1*; -2.5-fold change) and *HMGCS2* (-28.0-fold change) and may partly explain lower plasma BHBA after IMI challenge for this study. Concentration of milk BHBA were elevated at 12 and 36 h post-IMI challenge and returned to pre-challenge levels by 48 h (Figure 5C). Furthermore, yield of milk BHBA increased at 12 h post-IMI challenge (Figure 5D) and indicates that the lower BHBA observed in blood is due to an increase transfer into milk after IMI challenge.

Changes in fglu, BHBA, G6P, lactose, citrate and fat in milk relative to IMI challenge are shown in Figure 5. In addition, milk protein % decreased during IMI challenge (data not shown). Yield of fglu, lactose, fat, protein (data not shown) and citrate were lower whereas yield of G6P and BHBA were higher after IMI challenge. Decreases in milk component yield are mostly explained by lower milk yield (-36% by d = 2 post-challenge) observed when compared to the pre-challenge period. Transcriptomic-level profiling of mammary quarters at 24 h post-IMI challenge revealed no changes in key genes associated with glucose metabolism and utilization between challenged and unchallenged quarters [19] and therefore cannot support changes in major milk components discussed in this study. Concentration of citrate was greater by 36 h post-IMI challenge followed by a decrease in citrate to concentrations below those observed at h = -12 (Figure 5I) whereas citrate yield was lower by 24 h post-IMI challenge (Figure 5J). Concentration of milk citrate was also shown to decrease in LPS challenged quarters from lactating dairy cows [42]. Milk citrate, a marker of mitochondrial metabolism in the mammary gland [43], induces the ferric citrate transport system and is competing with lactoferrin for iron [42]. Lactoferrin contributes to host defense by binding iron thereby reducing availability of iron to invading bacteria [44] and lower yield of citrate may indicate an increase in the iron-binding capacity of bovine lactoferrin [45].

Concentration and yield of milk lactose decreased by 24 h after IMI challenge and returned to pre-challenge levels by 72 h (Figures 5G and H, respectively). Lactose is the major osmole in milk and decreases in milk yield and the synthesis milk components, such as lactose and protein, most likely explain the majority of changes in lactose during IMI challenge. However, lower milk lactose yield may be attributed to lower yield of fglu (Figure 5B where the yield of fglu (Figure 5B) was lower at 24 and 48 h when compared to -12 h relative to IMI challenge. Both concentration and yield of G6P (Figure 5E and F, respectively) rose after IMI challenge. Elevated levels of G6P by 24 h post-IMI challenge may signify increased conversion of fglu from lactose synthesis and towards the synthesis of G6P. However, this is not supported by the transcription-level profiling in mammary tissue at 24-h after IMI challenge [19]. Glucose-6-phosphate may serve as a substrate for the pentose phosphate pathway for the production of reducing equivalents used for several anabolic processes [24].

## Conclusions

Although drops in feed intake and milk yield are major contributors to changes in the metabolic response in blood and milk, the early rise in plasma NEFA during IMI challenge with *E. coli* may be partly attributed to increased adipose tissue lipolysis. Lower plasma BHBA may be associated with increase transfer into milk. We are the first to characterize changes in fglu and G6P in milk during IMI challenge. Lower yield of milk lactose may be attributed to lower yield of fglu. Higher G6P yield after IMI challenge may signify increased conversion of fglu to G6P. Results identify the metabolic response of various parameters in blood and milk and characterize the changes in fglu and G6P after IMI challenge with *E. coli* for cows in early lactation that may partly explain the partitioning of nutrients and changes in milk components in dairy cows with mastitis during early lactation. Future research is needed to determine how i.e. stage of lactation, parity, bacteria alter these metabolic changes that may help identify risk factors for the development, severity and duration of mastitis for dairy cows during lactation.
